# Among Adolescents, BDNF and Pro-BDNF Lasting Changes with Alcohol Use Are Stage Specific

**DOI:** 10.1155/2020/3937627

**Published:** 2020-01-25

**Authors:** Maria Jose Miguez, Diego Bueno, Luis Espinoza, Wenyaw Chan, Caroline Perez

**Affiliations:** ^1^School of Integrated Science and Humanity, Florida International University, 11200 SW 8th Street, ECS 450, Miami, FL 33199, USA; ^2^Miller School of Medicine, University of Miami, 1600 NW 10th Ave #1140, Miami, FL 33136, USA; ^3^Department of Biostatistics, University of Texas Health Science Center, 7000 Fannin St #1200, Houston, TX 77030, USA

## Abstract

Adolescent alcohol use demonstrates distinct developmental trajectories with dissimilar times of onset and trajectories. Given the importance of brain-derived neurotrophic factor (mature BDNF) in this development stage, the current study investigated its relationship with alcohol use. It also extends the literature by assessing the role of its precursor (pro-BDNF). Therefore, over the span of 5 years, we enrolled and followed participants to define age-related changes in BDNF levels in healthy adolescents. Then, the onset and frequency of alcohol use from ages 11 to 18 were collected to determine how the relationship between alcohol, pro-BDNF, and m-BDNF unfolds over time. With respect to development, analyses demonstrated that BDNF concentration slowly increases throughout adolescence. However, despite having similar basal BDNF levels, compared to controls, adolescents that started drinking before 15 years of age always exhibited lower BDNF levels. They also had a significant decrease in pro-BDNF levels. On the other hand, levels of mature BDNF steadily increased (974.896 ± 275 pg/ml) in those starting alcohol use after the age of 15. Similar to the younger users, a significant drop in pro-BDNF levels was observed over the course of the study. Our results suggested that both pathways may participate in the complex processes of alcohol dependence. The findings highlight the relevance of assessing alcohol-associated changes across the different phases of this vulnerable developmental period. This is the first study evidencing that m-BDNF changes associated with drinking behaviors differed between young and older adolescents. It is also the first article, documenting that drinking during adolescence leads to long-term decreases in pro-BDNF. These results have important implications for policies and programs targeting alcohol use disorders.

## 1. Introduction

Developmental studies have recognized the critical role of brain-derived neurotrophic factor (BDNF), in the process of brain development [1, 2]. BDNF is responsible for the structural and functional maturation of key areas of the brain such as the hippocampus and the frontal cortex [1–3]. Studying the effects of neurotrophins during the developmental period in animals has been useful to understand the roles of BDNF in branching, synaptic differentiation, pruning, and structural changes. However, information on humans is still limited [1–3].

Beyond promoting neuronal survival and its neurotrophic properties, animal studies have started to explore the contribution of BDNF to the development and trajectories of alcohol use disorders [4, 5]. Researchers have found that *BDNF* is implicated in the initiation of alcohol use, and its expression is enhanced in those freely consuming low doses of alcohol [6, 7]. On the other hand, when *BDNF* is blocked, animals increase both anxiety and alcohol consumption [8]. Evidence also suggests that *BDNF* may decrease the rewarding effects of alcohol, reducing alcohol-induced neuronal losses and depression by regulating serotonin and altering tryptophan concentrations [8–10]. BDNF can also alter the dopaminergic system in brain areas involved in alcohol dependence [11, 12]. Additionally, *BDNF* has been shown to alter cognitive performance relevant to decision-making and addiction [12–15]. However, the few human studies in existence are limited to adults and thus are not applicable to adolescents. Moreover, the widespread use of alcohol throughout adolescence requires a developmental approach to study its onset and trajectories.

Similar to other growth factors, BDNF is initially synthesized as a precursor (pre- and pro-BDNF protein isoforms). Then, the 32 kDa pro-BDNF is processed either intracellularly or in a synaptic space to produce mature BDNF (via the Golgi system) or is secreted as pro-BDNF without any modification [16, 17]. Data suggests that the length of the predomain influences this outcome; specifically, the longer versions favor the secretion of the immature isoform [16, 17]. This fine-tuned equilibrium is of relevance, as prior algorithms assumed that propeptides did not have a physiological function until the study done by Yang and colleagues [16]. Moreover, evidence supports a “yin-yang effect,” in which pro- and m-BDNF elicit opposite biological effects by activating two distinct receptor systems. Of relevance, the expression of pro-BDNF and p75NTR in animal models is developmentally regulated and appears to coincide with the development of risky behaviors [18, 19].

Of interest, pro-BDNF and m-BDNF play opposite effects. While m-BDNF promotes neuronal survival and growth, pro-BDNF can induce neuronal apoptosis via binding with p75 neurotrophin receptor (p75NTR) and sortilin [20]. We do not know much about the regulation between the central and the peripheral levels of BDNF. So far, data indicates that BDNF is released to the jugular vein and platelets uptake some of this amount. It has been elegantly demonstrated that the levels of BDNF in serum mirror those in washed platelet lysates [21]. On the other hand, plasma seems to reflect brain homeostasis [22]. In healthy adults, plasma BDNF levels are widely variable 8.0–927.0 pg/ml with a mean of 92.5 pg/ml and decreased with advancing age in both genders [23, 24]. However, those studies have sample sizes between 11 and 72 subjects. Unfortunately, no specific data exist in adolescents, further complicating the interpretation of results.

Expanding current knowledge is critical given that national statistics estimate that there are over 10 million underage drinkers in the United States [25]. Therefore, there is a need to extend current knowledge on key factors relevant to this developmental period that can be used as biomarkers or as targets for therapy. BDNF serves as a neurotrophic factor and regulates neurotransmission and plasticity so the BNDF pathway is a relevant candidate, yet there are still several knowledge gaps that need to be fulfilled, for example, establishing the dynamics of BDNF and pro-BDNF along the continuum of brain development. Since the old commercial technology does not differentiate between the pro- and mature isoforms of the polypeptide, the role of pro-BDNF in this setting is still unknown.

Therefore, the main goal of this translational research is to investigate the bidirectional relationship of m-BDNF and pro-BDNF and adolescent alcohol intake. Such analyses are critical to increasing our knowledge of neurobiological factors underlying both vulnerability and progression to dependence. The opposing nature of these peptides prompted us to hypothesize that pro-BDNF and m-BDNF might serve as punishment and reward signals in alcohol use behaviors. However, once alcohol use is established, an adaptive neurotrophic response is triggered.

## 2. Methods

“ROBIM” (the Role of Brain Derived Neurotrophic Factor in Decision Making Participants) is a 5-year longitudinal study based in South Florida. This article focuses on the responses of the 400/500 Hispanic adolescents that have completed follow-ups, which the American Academy of Pediatrics defines as minors (11 to 18 years old). Ethnicity was determined using participants' self-identification, country of origin, and the ethnicity of the parents and grandparents.

Adolescents were recruited through direct outreach in centers that provide recreational, social, and educational services for Hispanics and in health care facilities. Adolescents were ineligible if they had a history of a major neurological/psychiatric disorder (i.e., autism, severe developmental problems, mental retardation, and schizophrenia) or clinical disease (i.e., cancer and renal or heart disease) or receiving any neuropharmacological intervention or taking bodybuilding substances (i.e., steroids and growth hormones). Participants were consecutively enrolled between January 2012 and July 2015 from South Florida, US.

After a complete description of the study to the adolescent and his/her legal guardian, written informed consent was obtained from both for all minors. Only 25 adolescents were invited to participate but were not enrolled because of schedule conflicts.

### 2.1. Assessments and Follow-Ups

ROBIM's baseline visit was conducted by trained interviewers and consisted of a brief medical exam, structured survey questionnaires, and a blood sample to test BDNF levels. As part of the protocol, parents completed short questionnaires regarding sociodemographics and adolescents' health, including exposure to alcohol or drugs during pregnancy and family history. Consented adolescents and parents were interviewed separately.

Participants were followed longitudinally and assessed at baseline and 12 and 24 months. The annual visits mirrored the baseline assessments.

### 2.2. Alcohol Drinking

At each visit, participants were asked about alcohol intake in the past 6 and 12 months using the Alcohol Dependence Scale (ADS) [26]. Participants were asked to report alcohol consumption using glass models of 12 ounces of beer, 5 ounces of wine, or 1.5 ounces of liquor. Alcohol consumption scores were computed by averaging cross products of quantity and frequency of beer/wine and hard liquor. Then, as recommended by national guidelines, we used the number of binge drinking episodes as a marker for hazardous use. Binge drinking was defined as the consumption of alcohol sufficient to elevate blood alcohol concentration to 0.08; commonly, this concentration is equivalent to 3 drinks for boys aged 9-13, 4 drinks for those aged 14-15, and 5 drinks for those boys aged 16 or older. The rate for girls between ages 9 and 17 was 3 drinks.

Participants were asked if any member of the family, particularly close ones, had an alcohol abuse problem. If the answer was yes, we asked them to identify who it was.

### 2.3. Precursor and Mature Brain-Derived Neurotrophic Factor (BDNF) Concentrations

Circulating levels of BDNF were selected because prior studies have demonstrated that although different from levels in cerebrospinal fluid (CSF), they are correlated with CSF measures in other central nervous (CNS) diseases [18]. To obtain platelet-poor plasma, blood samples were collected in EDTA-coated tubes between 8 and 11 a.m. to minimize effects of a circadian rhythm and were stored on ice. Plasma was separated by centrifugation at 40°C for 15 min at 1500 × *g*. This plasma was again recentrifuged at 10000 × *g*, and aliquots of PPP were stored until assayed.

Pro-BDNF concentrations were established according to the manufacturer's directions using NovaTeinBio proBDNF Rapid enzyme-linked immunosorbent assay (Minneapolis, MN, USA). Levels were expressed in pg/ml.

BDNF concentrations were quantitatively determined using MILLIPLEX MAP Human Pituitary Magnetic Bead Panel from Millipore (EMD Millipore Corporation, Billerica, MA, USA). All m-BDNF and pro-BDNF measurements were performed in duplicate and following the manufacturer's procedures. Their concentrations were expressed in pg/ml.

### 2.4. Control Variables

Information was collected on potentially confounding variables, including adolescent/parent sociodemographics (age, education, employment, gender, and country of birth) and medical history. We gathered information on variables known to affect BDNF, such as other abused drugs, exercise (Stanford 7-day survey), and body mass index [27].

### 2.5. Statistical Analyses

Descriptive statistics were used to summarize the data. Group comparisons were assessed using the chi-square test for categorical variables, ANOVA for normally distributed variables, and the Wilcoxon rank sum test for nonparametrically distributed continuous variables. Correlation analyses between variables of interest were performed using Pearson's correlation coefficient (*r*). The General Linear Model (GLM) was applied to examine the relationship between alcohol drinking and m-BDNF and pro-BDNF during the length of the study. Another logistic regression model was employed to identify predictors of early alcohol use (e.g., onset drinking before 15 years old).

## 3. Results

### 3.1. Sample Characteristics

The demographic characteristics of the adolescents enrolled in ROBIM are shown in Table 1. The male : female ratio was nearly 1 : 1. The age of the respondents ranged from 11 to 18 years (14.6 ± 2.3 years). According to the development stages of adolescence, the majority of participants were in either early (11–14 years old (45%)) or middle (15–17 years old (*n* = 30 subjects)) adolescence, with the remaining participants being on the late phases of adolescence (>18 years old (25%)). The study successfully recruited a sample of both high- (40% < 80000) and middle/low-income participants. Most adolescents were native-born Americans, and 27% were foreign-born.

**Figure 1:**
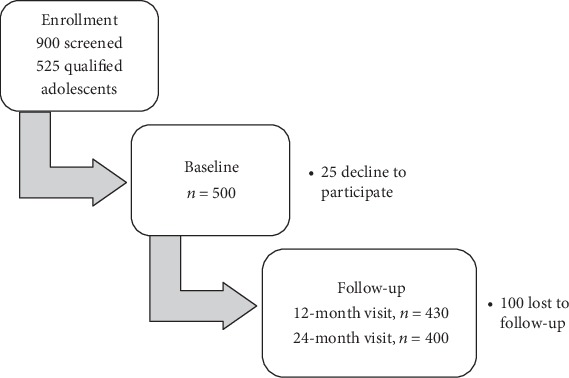
ROBIM study overview.

Table 1 and Figure 1 provide information on those lost to follow-up. As illustrated in the table, compared to those adherent to the study visits, those lost due to attrition were slightly older. Other than that, they did not differ in other sociodemographic or behavioral characteristics. Notably, the majority were lost during the first year of follow-up, after that only 30 adolescents missed the second-year visit. The main reason was migration out of the state or parents' conflict with the schedule. Only 5% expressed no interest on returning because they had other activities.

### 3.2. Alcohol Use

At the time of enrollment, 76% of the sample had never tasted alcohol and the remaining 24% had at least one drink of alcohol on one or more days during their life. Overall, 24% of the sample reported using alcohol in the past six months. On average, these adolescents started drinking when they were 16 years of age (2 SD, national average = 14); however, 16% of our sample had their first drink before the age of 13. No significant gender differences in the age of alcohol initiation were observed.

### 3.3. Developmental Analyses

In contrast to adult patterns of daily use, adolescents were more likely to drink and binge drink in social groups. We observed a normative increasing trajectory with younger adolescents reporting drinking 2.4 ± 1 drinks, those aged 15-17 years consuming approximately three drinks per day, and those > 17 years consuming an average of almost 4 drinks per occasion (3.5 ± 1.7, *p* = 0.1).

Their alcohol use steadily increased in all groups, but much less in the older ones, as compared to the middle and the young ones (0.2 ± 0.2 (*p* = 0.5) vs. 2.2 ± 1.2 (*p* = 0.08) and 1.8 ± 1.3 drinks (*p* = 0.3) in those < 15 years). Increases in alcohol use slightly differed by gender, with females' drinking barely increasing overtime (0.3 ± 0.2, *p* = 0.3). On the other hand, males increased their consumption by two additional drinks per occasion from baseline to the end of the study (1.8 ± 1 drinks, *p* = 0.1).

Alcohol consumption in terms of maximum consumption per occasion was highly variable, going from one to twenty-three drinks. The average consumption for males was 7.4 ± 5.1, which was not different than the consumption of alcohol among females (4.5 ± 3.3).

The percentage of people aged 15 or younger reporting binge drinking was 6% and rises to 16% in those aged 17 years and older. Over time, some of the adolescents (21%) began to drink heavily—consuming four or five drinks per occasion, two or three times a month—classic binge drinking behavior in teenagers.

### 3.4. BDNF Longitudinal Analyses: Developmental Trajectories

To determine which changes in BDNF levels represent a pathological change, it is necessary to analyze the average adaptive changes during this time of brain development. There was a negative correlation between plasma BDNF levels and age (*r* = −0.20, *p* < 0.05). Levels range from 27 to 23047 pg/ml, but the mean of the healthy adolescents that never used alcohol or drugs was 2324 ± 349.8 pg/ml. Based on prior literature, we explored gender differences regarding BDNF levels, but differences were not evident.

Then, stages of adolescence were used for these analyses: a one-way ANOVA of BDNF levels demonstrated significantly different levels among the three groups. Pro-BDNF levels fluctuated between 436 and 17923 pg/ml (3832.8 ± 2280). In contrast to m-BDNF, levels were similar across age groups. There were also similar levels between male and females, though values tended to be lower in the latter group.

**Figure 2:**
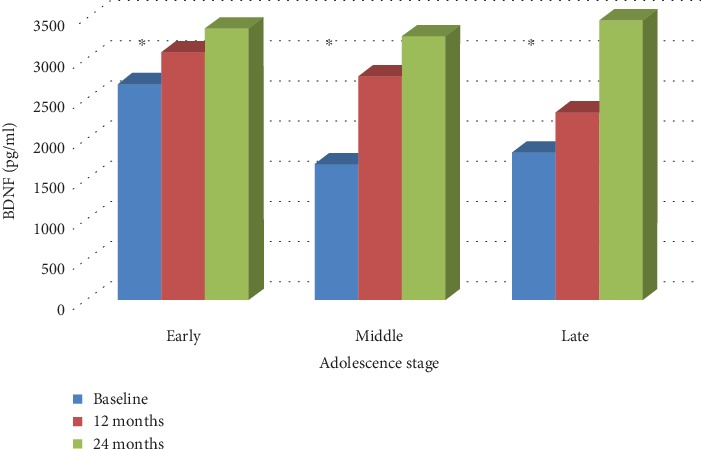
m-BDNF levels by the adolescence stage. Changes in mature BDNF levels with age. ∗ points to significantly different values (*p* = 0.04).

As depicted in Figure 2, the BDNF concentration increased over the pubertal years. The youngest exhibited significantly higher values. On the contrary, pro-BDNF significantly increased and peaked around eighteen. These data indicate that further analyses need to consider the age/developmental stage of the participant.

### 3.5. Pro-BDNF, BDNF, and Alcohol Use

Next, we analyzed neurotrophic changes linked with alcohol use. A significant association was found between the level of alcohol consumption and BDNF levels in those individuals who reported any level of alcohol consumption at baseline. Correlation analysis showed that alcohol initiation was correlated with pro-BDNF levels (*r* = −0.20, *p* = 0.001).

**Figure 3:**
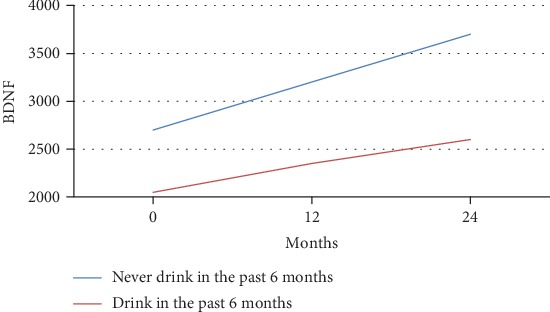
BDNF by the drinking group for adolescents younger than 15 years of age. As depicted, the analysis shows significant changes during time within each group within a linear mixed model. Nondrinkers under 15 experienced a sharper incline in m-BDNF level than drinkers.

**Figure 4:**
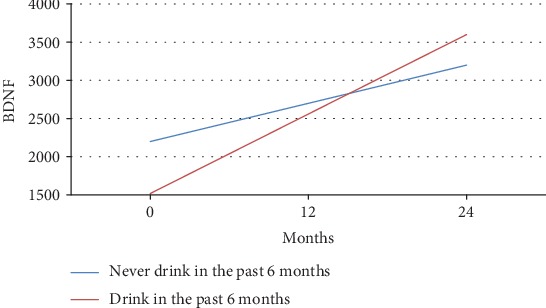
BDNF trend of changes during the study in adolescents > 15 years. Change in m-BDNF by the drinking group for adolescents over 15 years of age. Both drinkers and nondrinkers experienced inclines in m-BDNF, but the slope of changes for drinkers was more intense.

The main effect of lifetime alcohol use on BDNF concentrations was observed. This effect resulted in decreased levels of BDNF in the alcohol group compared with those measured for the control group (see Figures 3 and 4).

Since prior studies have demonstrated that subjects who used alcohol before the age of 15 are at an increased risk of alcoholism, risky behaviors, and underachievement, we used this cutoff point to proceed with further analyses.

Figure 3 depicts the mean BDNF over time separated by alcohol users and nonusers for adolescents aged 15 years or younger. These models were based on longitudinal regression analysis where alcohol use (*p* = 0.50), time (*p* = 0.24), and their interaction (*p* = 0.41) were not significant.  Figure 4 illustrates the mean BDNF over time separated by alcohol users and nonusers for adolescents over 15 years of age. The slope was significantly different (2171.35 pg/ml vs. 2590.1 pg/ml).

**Figure 5:**
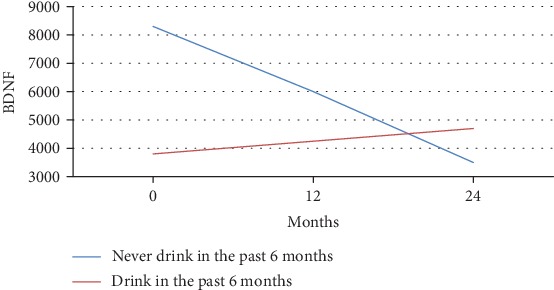
Pro-BDNF by the drinking group for age ≤ 15. Representation of m-BDNF levels at different time points and the trend of changes during the study period. Younger adolescents that did drink in the previous 6 months experienced a significant decline in pro-BDNF. Nondrinkers had slight increases over time.

**Figure 6:**
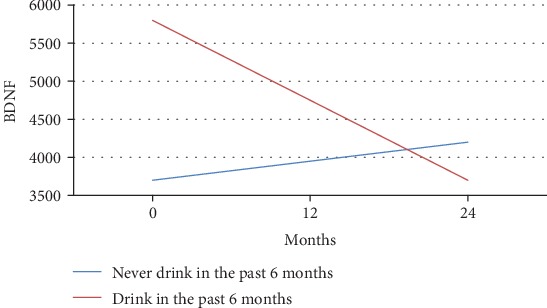
Pro-BDNF by the drinking group for age > 15. Pro-BDNF levels at different time points in older adolescents by the drinking group. Older adolescents mimicked the trend of those under 15 years of age. Drinkers experienced declines in pro-BDNF, and nondrinkers had a steady increase in pro-BDNF over time.

As most features of addiction develop progressively due to repeated exposure to alcohol, we analyzed the effect of alcohol use and pro-BDNF over the following 24 months. As depicted in Figures 5 and 6, pro-BDNF was significantly higher in those consuming alcohol (6089.3 ± 826 vs. 4056.4 ± 2880 pg/ml, *p* < 0.000).

Overall, analyses indicated that alcohol use is accompanied by a significant decrease in pro-BDNF levels and an increase in mature BDNF over time, which suggests altered regulation of pro-BDNF proteolytic processing.

## 4. Discussion

Adolescence is a developmental period characterized by both neurological and risk-taking behavioral changes [1]. Among them, alcohol use remains as a major public health problem. Overall, alcohol use was frequent among our sample of adolescents. Underage alcohol use was reported by a third by the end of the study, a percentage similar to that in national reports [28]. Binge and heavy use also increase overtime. These findings emphasize the need to understand the underpinnings of this phenomenon in order to design science-based interventions to address this problem.

In this regard, our study also provides compelling data of the close relationship between BDNF and alcohol use in humans during this developmental stage. Changes in BDNF were evident after the adolescent engaged regularly in alcohol use, which probably reflects adaptive changes to chronic stress exposure (both to alcohol and to the environment). Other researchers have examined the association between BDNF and alcohol use. However, no other study has done a longitudinal investigation while considering the stage of adolescent development. In addition, many of them erroneously used serum instead of plasma.

Since BDNF has been mostly studied in animal models and the few human studies are in the context of pathological conditions (e.g., mood and psychiatric disorders) or based on its genes, homeostatic information is very limited [29–34]. Although genetic studies have been highly informative, epigenetic changes are equally relevant in this context. Therefore, measurements of the actual protein and not only the genetic background are necessary. To our knowledge, this is the first study demonstrating in humans that levels of BDNF are not constant during the adolescence period. Similar to animal models, we have found that BDNF protein levels change during the time of brain development, yet the slope of changes is different by the developmental stage [35]. Such changes may explain the discrepancies observed among studies focusing on adolescents, for example, those with physical activity.

Findings of low BDNF levels among those young adolescents who started drinking are in line with animal studies indicating that a reduced expression in BDNF (in heterozygous BDNF female mice or in CREB mice) correlates with an increased risk of alcohol consumption [36–38]. Multiple lines of evidence have further linked alcohol preference and dependence with lower expression of BDNF in the amygdala and the prefrontal cortex. It needs to be noted that baseline differences in BDNF were nonsignificant, yet those who started drinking before the age of 15 had significantly lower BDNF levels. Such a finding is highly relevant since BDNF is involved in regulating brain plasticity but also in mood, attention, motivation, and decision-making key in heightened risk-taking.

Contrary to the reduced BDNF concentration observed subsequent to alcohol exposure in young adolescents, the older counterpart exhibited increases under conditions of moderate to high alcohol intake. These rises in BDNF are similar to those observed in animals in which chronic exposure to either a 2-bottle choice of alcohol exposure or 2.0 g/kg alcohol injection results in increases in BDNF expression in the dorsal striatum, compared to age-matched alcohol-naïve mice. These upsurges can represent an effort to protect the brain against alcohol-induced cell damage, since BDNF controls dopaminergic, glutamatergic, and serotonergic neurotransmissions [36, 39]. Animal and preclinical models have documented that BDNF manipulations reverted those behaviors, implicating BDNF in the homeostatic regulation of various alcohol-related behaviors [40].

Equally important, this is the first analysis of the relationship between pro-BDNF and alcohol use development in humans. Analyses confirm the hypothesis that higher pro-BDNF levels are a precondition predisposing adolescents to “preference” and high alcohol intakes. However, data also indicates that the pro-BDNF/BDNF system undergoes significant changes as a result of hazardous alcohol consumption.

Generalization of the research data should be cautious because this study was limited to Hispanic adolescents, and thus, it is important to confirm results with other racial ethnic groups. It also needs to be noticed that the BDNF changes were assessed in circulation, and while some researchers may argue that brain specific measurements are the gold standard, it needs to be noticed that measuring BDNF levels in the brain is not only difficult but also impractical. While under the old concept that posit that the brain was isolated, central measures were relevant, studies have demonstrated that BDNF cross the blood-brain barrier in both directions [6, 41]. Studies demonstrating that peripheral and central BDNF levels are associated are further providing support to our approach [42].

## 5. Conclusion

To conclude, this is the first study evidencing that m-BDNF changes associated with drinking behaviors differed between young and older adolescents. We were also pioneers in defining whether changes mediated by pro-BDNF are operative in the regulation of BDNF and alcohol use in adolescence. Our analyses showed that pro-BDNF regulates BDNF production and has a significant relationship with alcohol use behaviors. It is also the first article, documenting that drinking during adolescence leads to long-term decreases in pro-BDNF. These results have important implications for policies and programs targeting alcohol use disorders.

## Figures and Tables

**Table 1:** Demographic characteristics of the adolescent population (*n* = 500).

Demographic variable	Nonalcohol user	Alcohol user	Lost to follow-up	*p* values
Gender				
Male	71%	29%		0.8
Female	75%	25%		
Age in years	14.5 ± 2.2	16.5 ± 1.4	15.4 ± 2.2	0.000
Education	8.2 ± 2.3	10.0 ± 1.6	9.3 ± 2.3	0.000
Income				
Low/poverty	30%	30%	33%	0.8
Middle	23%	25%	27%	
High class	47%	45%	40%	
Immigrant	77%	23%	72%	0.9
Born in the USA	76%	24%	28%	
Body mass index	23.3 ± 5.1	24.0 ± 6	23.7 ± 5.2	0.5

Values are means ± deviations or percentages. Significant differences were found for age and levels of education.

## Data Availability

The data used to support the findings of this study may be released upon application to the Florida International University IRB.
